# Influence of Diets Differing in Macronutrient Composition on Metabolic Regulation During Exercise in Adults with Type 1 Diabetes

**DOI:** 10.3390/nu17233637

**Published:** 2025-11-21

**Authors:** Olivia Mary McCarthy, Kasper Birch Kristensen, Ajenthen Gayathri Ranjan, Chloe Nicholas, Jens Juul Holst, Richard Michael Bracken, Kirsten Nørgaard, Signe Schmidt

**Affiliations:** 1 Copenhagen University Hospital—Steno Diabetes Center Copenhagen, 2730 Herlev, Denmark; kasper.birch.kristensen.01@regionh.dk (K.B.K.); ajenthen.ranjan@regionh.dk (A.G.R.); kirsten.noergaard@regionh.dk (K.N.); 2 Applied Sport, Technology, Exercise and Medicine Research Centre, Faculty of Science and Engineering, Swansea University, Swansea SA2 8PP, UK; chloe.nicholas@regionh.dk (C.N.); r.m.bracken@swansea.ac.uk (R.M.B.); 3 Novo Nordisk Foundation Center for Basic Metabolic Research, Faculty of Health and Medical Sciences, University of Copenhagen, 2200 Copenhagen, Denmark; jjholst@sund.ku.dk; 4 Department of Biomedical Sciences, Faculty of Health and Medical Sciences, University of Copenhagen, 1172 Copenhagen, Denmark; 5 Health Technology and Solutions Interdisciplinary Research Institute, Faculty of Science and Engineering, Swansea University, Swansea SA2 8PP, UK; 6 Department of Clinical Medicine, Faculty of Health and Medical Sciences, University of Copenhagen, 1172 København, Denmark

**Keywords:** type 1 diabetes, exercise, metabolism, diet, nutrition, physiology

## Abstract

**Aim:** To compare the effect of consuming three isocaloric diets that differed in macronutrient composition on substrate oxidation and glucose regulation during sustained submaximal exercise in adults with type 1 diabetes (T1D). **Methods:** In a randomised, crossover design, 12 adults with T1D (n = 4 female, age: 46 ± 15 years, HbA1c: 55.9 ± 7.8 mmol/mol) consumed three isocaloric diets over seven days: (i) HCLFLP (high-carbohydrate [48%], low-fat [33%], low-protein [19%]), (ii) LCHFLP (low-carbohydrate [19%]), high-fat [62%], low-protein [19%]), and (iii) LCLFHP (low-carbohydrate (19%), low-fat [57%], high-protein [24%]). On the morning of day eight, participants undertook 45 min of cycling (≈60% $\dot{V}$O_2peak_) whilst fasting. Venous-derived plasma glucose and free fatty acids (FFA) were measured throughout the trial period. Indirect calorimetry was used to determine rates of substrate oxidation during exercise. Data were analysed via repeated measures ANOVAs with *p* ≤ 0.05 accepted as significant. **Results:** During exercise, rates of lipid oxidation were higher (1.2-fold, *p* = 0.030) and carbohydrate oxidation lower (0.8-fold, *p* = 0.030) in LCHFLP versus HCLFLP. Concentrations of FFA after exercise were higher in LCHFLP compared to HCLFLP (by ≈22%, *p* = 0.019). Overall time spent in euglycaemia was higher (HCLFLP: 55.6 ± 43.9, LCHFLP: 87.3 ± 28.7, LCLFHP: 95.2 ± 7.9%, *p* = 0.003) and hyperglycaemia lower (HCLFLP: 44.4 ± 43.9, LCHFLP: 12.7 ± 28.7, LCLFHP: 4.8 ± 7.9%, *p* = 0.003) in both LC diets relative to HC. No differences in any measured biomarkers were observed between the two LC diets. **Conclusions:** One-week consumption of isocaloric diets that differed in their macronutrient composition shifted patterns of energy metabolism during a standardised bout of moderate intensity exercise performed in the fasted state in adults with T1D.

## 1. Introduction

Although endorsed by leading health organisations as a key component of the optimal management plan of those with type 1 diabetes (T1D) [1,2], exercise often represents a glycaemic challenge. Contrary to the endogenous regulation of glucose homeostasis in those without the condition, individuals with T1D are reliant on exogenous insulin therapy (which is not under physiologic control) to manage glycaemia. In the context of physical exercise, the additive effects of hyperinsulinaemia and exercising muscle tissue can result in marked, and often clinically concerning, drops in blood glucose [3,4,5]. With this in mind, current consensus guidelines for exercise management focus on reducing insulin on board and consuming carbohydrates around exercise to help keep glucose within a near-normal range [6,7].

By virtue of their primary role in determining glucose metabolism, carbohydrates, and their resultant relationship to exogenous insulin therapy demands in the context of T1D have taken precedence as the point of dietary focus in many exercise-centric research studies. Consequently, far less attention has been paid to the other macronutrients i.e., *fat* and *protein* and whether alterations in their relative contribution to overall energy intake influence energy metabolism during exercise in those with T1D. This is rather surprising given the recognised mediatory role of nutritional status in determining substrate availability, glycaemia, and intramuscular fuel utilisation during physical exercise in the general population [8,9,10].

Diets high in carbohydrates maintain or raise muscle and liver glycogen content resulting in a continued contribution of carbohydrates to intramuscular energy supply during exercise. Conversely, consuming a fat-rich diet increases triacylglycerol content in skeletal muscle, raises plasma fatty acid concentrations, and promotes higher rates of fat oxidation during exercise [11,12]. Shifts in fuel metabolism occur reciprocally, such that diets high in fat downregulate carbohydrate metabolism during exercise, even after acute restoration of carbohydrate availability [13]. Notably, these dietary-induced intramuscular metabolic adaptations can occur in a relatively short time frame, e.g., five days [14,15,16,17].

Normally, exercise performed in the fasted as opposed to the fed state leads to both a larger degradation of endogenous glycogen utilisation and an increased reliance on lipid for energy turnover [18]. In people without T1D, the carbohydrate-induced increase in plasma insulin concentrations exerts a potent inhibitory effect on lipolysis and consequently reduces fat oxidation during exercise whilst promoting intramuscular glucose uptake and utilisation. Various studies have shown that T1D is associated with impaired and maladaptive metabolic responses to exercise, including abnormalities in substrate utilisation [19,20,21,22,23,24]. Yet, the majority of these investigations have concentrated on cohort comparisons against healthy controls where the impact of diet was not the focal point. Furthermore, much of our understanding of energy metabolism during exercise in T1D stems from studies undertaken in the post-prandial state, with an emphasis on how peri-exercise insulin dose adjustments effect glycaemic safety outcomes.

We have previously shown that compared with consuming a high-carbohydrate, low-protein meal acutely (1.5 h) before commencing cycling, one that was instead low in carbohydrate and high in protein, improved glucose variability and lessened the drop in glucose during exercise [25]. In extension of these acute pre-exercise dietary manipulations, we more recently showed that diets low in carbohydrate but high in fat or protein content over seven-days was associated with lower glycaemic variability than an isocaloric high-carbohydrate, low-fat, low-protein diet in adults with T1D [26]. However, unaccounted for in this study was the potential implications of these short-term alterations on energy metabolism during exercise in people with T1D.

With this in mind, this study compared the effect of consuming three isocaloric diets that differed in macronutrient composition on substrate oxidation and glucose regulation during sustained submaximal exercise in adults with T1D.

## 2. Methods and Materials

### 2.1. Study Design and Ethical Approval

This was a randomised, three-period, crossover study during which participants adhered for seven days to three diets that each differed in their macronutrient composition before attending the laboratory for the exercise experiment. This was a feasibility study and so the sample size was based off pragmatic capabilities. The study was approved by the Regional Committee on Health Research Ethics (H-21042230) and the Danish Data Protection Agency (P-2021-826), carried out in accordance with the principles of the Declaration of Helsinki, and registered at Clinicaltrials.gov (registration no. NCT05268705).

### 2.2. Screening Procedures and Eligibility Criteria

Participants were recruited from Steno Diabetes Centre Copenhagen, Denmark, and screened for eligibility after having provided informed consent. Main inclusion criteria were as follows: age ≥ 18 years, duration of T1D ≥ 5 years, insulin pump use ≥ 1 year, use of intermittently scanned or continuous glucose monitoring ≥ 3 months, HbA1c ≤ 8.5% (≤69 mmol/mol), self-reported hypoglycaemia awareness, and exercising for at least 30 min at a moderate or vigorous intensity twice per week. Main exclusion criteria were as follows: using drugs affecting glucose metabolism (other than insulin) during the study or within 30 days prior to study start, use of an automated insulin delivery system, ischemic heart disease, severe asthma, pregnancy, or breast-feeding.

At screening, data on diabetes characteristics, anthropometrics, blood pressure, and heart rate were collected. Eligible participants were subsequently randomised using the electronic data capture system, REDCap (Version 13.1.27, Vanderbilt University, Nashville, TN, USA).

### 2.3. Composition of the Diets

Three diet plans were developed by a registered dietitian for each participant based on the official Danish Dietary Guidelines [27], individual preferences, and energy needs. The macronutrient contents of the diets were defined according to a fixed amount of carbohydrate (e.g., high fat [HF] and high protein [HP]: maximum 100 g/day, high carbohydrate [HC]: minimum 250 g/day) and protein (HC and HF: 1.4 g/kg body weight/day, HP: 1.8 g/kg body weight/day). Fat content was then calculated as the remaining quantity needed to achieve an isocaloric energy intake across the three diets (Table 1 shows an example of the macronutrient composition of the diets for a 70 kg person). The HC and HF diets were characterised by a relatively high protein content, while the HP diet had a relatively high fat content, albeit lower than that of the HC and HF diets, respectively (please see Table 1). Each plan allowed participants to substitute dishes with alternatives, providing dietary variation while still adhering to the diet criteria.

The diets were hence referred to as the following throughout the remainder of text; HCLFLP; high carbohydrate, low fat, low protein, LCHFLP; low carbohydrate, high fat, low protein, and LCLFHP; low carbohydrate, low fat, high protein. There was a washout period of 5–35 days (during which there were no specified diet restrictions) between each weekly dietary intervention. Through their involvement in the study, participants were instructed to handle cases of hypoglycaemia (sensor glucose [SG] < 3.9 mmol/L) outside of the laboratory visits according to their usual treatment procedures. Participants were also asked to log any treatment carbohydrates in the total daily carbohydrate count and reduce their meal carbohydrate content in order to meet the carbohydrate limits. Consistency in activity levels was encouraged among participants throughout all three intervention periods. The deviations between the carbohydrate recordings in the insulin pumps as well as the planned carbohydrate intake was minimal (mean [IQ] HCLFLP: 4.8 [25.3; 9.9], LCHFLP: +10.7 [−3.1; 28.4], LCLFHP: +5.7 [−1.2; 2.5] g, *p* = 0.14) suggesting acceptable adherence to all three study diets. Further, physical activity levels assessed by daily energy expenditures (*p* = 0.70), metabolic equivalent of task rates (*p* = 0.70), and step counts (*p* = 0.90) did not differ between diet weeks. A more detailed overview of these data can be found in our previous publication [26] alongside example templates of each diet plan. 

### 2.4. Experimental Trial Day Procedures

Participants were encouraged to refrain from any intensive exercise for 48 h prior to each visit and to arrive at the research facility in the morning following an overnight fast with a blood glucose level of 7–10 mmol/L, which was the start exercise plasma glucose (PG) target. To achieve the target, minor insulin or carbohydrate corrections were permitted. If participants had insulin pumps with low glucose suspension or predicted low glucose management function, it was turned off during the study visits. Except for the preceding dietary intervention, the study days were identical. After confirmation of suitability to commence experimental procedures, an intravenous catheter was inserted into an antecubital vein for blood sample collection. Initially, the participants rested for 90 min. Subsequently, they cycled for 45 min at 60% of their $\dot{V}$O_2peak_ on an ergometer cycle (Lode Corival CPET, Groningen, The Netherlands). To ensure an exercise intensity at 60% of $\dot{V}$O_2peak_ breath-by-breath data were recorded using Vyntus (Vyntus, Vyaire Medical Inc., Chicago, IL, USA) integrated with heart rate information via chest belt telemetry (Polar, H10, Kempele, Finland). Spirometry data were processed using SentrySuite^TM^ Solution (Version 3.0, Vyaire Medical Inc., Chicago, IL, USA). Raw cardiopulmonary data were exported with 5 s intervals (SentrySuite^TM^ software, Vyaire Medical, Chicago, IL, USA) [28] and subsequently averaged in 30 s segments for statistical processing. Whole body rates of carbohydrate (CHO) and fat oxidation (in g/min) were calculated from $\dot{V}$O_2_ and $\dot{V}$CO_2_ values measured during each period of respiratory gas data capture throughout the trial day. Calculations were made with non-protein respiratory exchange ratio (RER) values being assessed according to standard equations [28]:

$$\text{CHO oxidation}=4.55\times \dot{V}{\mathrm{CO}}_{2}-2.21\times \dot{V}O_{2}$$

$$\text{Fat oxidation}=1.67\times \dot{V}O_{2}-1.67\times \dot{V}{\mathrm{CO}}_{2}$$

Finally, the participants rested and were observed for 90 min or until hypoglycaemia (PG < 3.9 mmol/L) or if they experienced unbearable symptoms of hypoglycaemia. Basal insulin was reduced by 50% from the beginning of the study visit (90 min prior to exercise) until the end of exercise according to international guidelines [6].

### 2.5. Blood Sampling Procedures

Venous blood samples were obtained in 5-to-30 min intervals over the 225 min trial period. Upon collection, 300 µL of whole blood was aspirated and processed immediately to determine point concentrations of PG and lactate (PLa) (YSI Inc., Yellow Springs, OH, USA). Following centrifugation, the remaining volume was stored at −80 °C for later analyses. Plasma glucagon concentrations were determined via a sensitive C-terminal specific glucagon radioimmunoassay [29]. Concentrations of non-esterified fatty acids (NEFA) were measured by Cobas 800 analyser, module C502 (Roche Diagnostics International Ltd., Rotkreuz, Switzerland) using a Wako NEFA-HR; 436-91995 (2) reagent (FUJIFILM Wako Chemicals GmbH, Neuss, Germany). Serum insulin was measured by Mercodia Iso-Insulin Elisa (10-1128-01, Mercodia AB, Uppsala, Sweden).

### 2.6. Statistical Analyses

All statistical analyses were performed via IBM^®^ SPSS^®^ Statistics (Version 29.0.1.0, Armonk, NY, USA, IBM Corp.). Descriptive statistics are presented as mean ± SD in text and tables and mean ± SEM in graphics. A two-way repeated measures ANOVA was run to determine conditional differences in hormonal and metabolic parameters across the acute trial day. In instances where sphericity was violated and the GreenHouse–Geisser ε value > 0.750, Huynh–Feldt results were reported. Interaction effects were stated if significant, otherwise main effects are referred to in text. In cases of the former, post hoc analyses to determine simple effects were conducted with Bonferroni adjustment to account for multiple comparisons over time. Between arm differences in point value metrics were determined via repeated measures ANOVAs with Bonferroni adjustment used for post hoc pairwise comparisons.

## 3. Results

### 3.1. Participant Characteristics

Baseline anthropometric, diabetes, and physical fitness characteristics of the 12 study participants (4 females) are displayed in Table 2.

Participants used a range of different insulin pumps: Medtronic ([Northridge, CA, USA] MiniMed 640G [n = 4]), Insulet ([Bedford, MA, USA] n = 6 Omnipod DASH), Tandem Diabetes ([San Diego, CA, USA] n = 1 t:slim X2), YPSOMED ([Burgdorf, Switzerland] n = 1 YpsoPump). Nine participants used insulin aspart whilst three used fast-acting insulin aspart (NovoNordisk, Bagsværd, Denmark) throughout the study period.

### 3.2. Metabolic and Hormonal Responses During Laboratory Trial Days

#### 3.2.1. Plasma Glucose Responses

**Figure 1:**
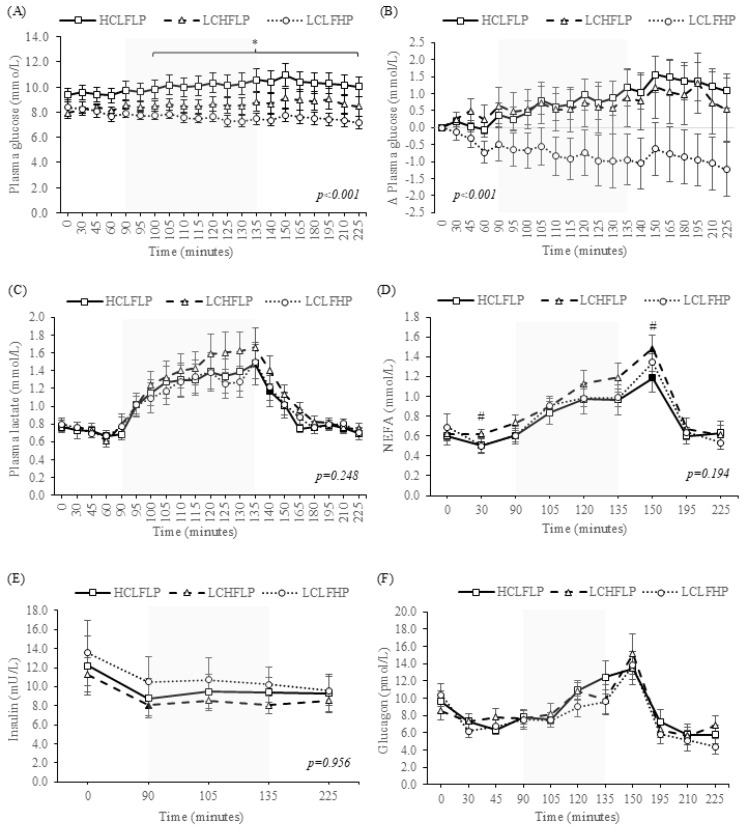
Acute metabolic ((**A**) [plasma glucose], (**B**) [plasma glucose when data are expressed as a relativised change from the respective baseline value], (**C**) [plasma lactate] and (**D**) [non-esterified fatty acids]) and hormonal ((**E**) [insulin] and (**F**) [glucagon]) responses across the acute experimental trial period. HCLFLP: The arm in which participants had consumed a high-carbohydrate, low-fat, low-protein diet in the seven days prior to the experimental trial visit. LCHFLP: The arm in which participants had consumed a low-carbohydrate, high-fat, low-protein diet in the seven days prior to the experimental trial visit. LCLFHP: The arm in which participants had consumed a low-carbohydrate, low-fat, high-protein diet in the seven days prior to the experimental trial visit. Grey shaded area denotes the time frame in which participants were undertaking the 45 min bout of moderate intensity continuous exercise programmed to be ≈60% of their individualised $\dot{V}$O_2peak._ The * denotes a significant difference (*p* ≤ 0.05) in the point concentration of a parameter between the HCLFLP and LCLFHP dietary arms. # denotes a significant difference (*p* ≤ 0.05) in the point concentration of a parameter between the HCLFLP and LCHFLP dietary arms. Black shaded markers denote a significant difference (*p* ≤ 0.05) in the point concentration of a parameter relative to the respective baseline value. Patterned shaded markers denote a significant difference (*p* ≤ 0.05) in the point concentration of a parameter relative to the respective pre-exercise, i.e., minute 90, value. *p* values displayed on figures denote the interaction effect level from the two-way repeated measures ANOVA. Data are presented as mean ± SEM.

Figure 1, panel B displays absolute PG concentrations across the 225 min trial period in all three dietary arms. A significant interaction effect between diet and PG levels over time was found (F [40, 360] = 2.539, *p* < 0.001). Main effects were found for both time (F [20, 180] = 2.211, *p* = 0.003) and diet (F [2, 18] = 7.574, *p* = 0.004); the latter accounted for in pairwise comparisons by the HCLFLP and LCLFHP arms (*p* = 0.030). PG concentrations differed between these two arms at all timepoints from minute 100 onwards (denoted via * in Figure 1, panel A).

The absolute exercise-induced change (Δ_Ex_) in PG differed between trial arms (HCLFLP: Δ + 0.72 ± 1.22, LCLFHP: Δ − 0.44 ± 1.42, LCHFLP: Δ + 0.23 ± 1.82 mmol/L, *p* = 0.039) driven by divergence between the HCLFLP and LCLFHP arms (*p* = 0.044). The same observations were noted in the rate of change in PG over exercise (HCLFLP: Δ + 0.02 ± 0.03, LCLFHP: Δ − 0.01 ± 0.03, LCHFLP: Δ + 0.01 ± 0.04 mmol/L/min, *p* = 0.038. Pairwise comparisons: HCLFLP vs. LCLFHP, *p* = 0.043) as well as the mean concentration of PG throughout the 45 min cycling session (HCLFLP: 10.1 ± 2.8, LCLFHP: 7.6 ± 1.2, LCHFLP: 8.6 ± 1.9 mmol/L/min, *p* = 0.004. Pairwise comparisons: HCLFLP vs. LCLFHP, *p* = 0.012) whereby the HCLFLP arm differed from that of the LCLFHP one. In no trial did PG change over exercise per se, i.e., PG levels at each stage of exercise remained comparable to those taken immediately prior to commencing cycling.

Figure 1, panel B displays PG levels across the trial period when data are expressed as a relativised change from baseline. Though a significant interaction effect was found (F [40, 360] = 2.539, *p* < 0.001), further investigation of simple effects revealed no between or within conditional differences.

Table 3 includes clinical glycaemic parameters during each pre-defined time-period on experimental trial days. With the exception of the isolated rested, pre-exercise period, the HCLFLP diet led to a higher PG mean and TAR with lower TIR than that of the LCLFHP arm in all other time-phases (all *p* ≤ 0.05, denoted via the * Table 3). The observed differences in TIR and TAR also extended to between the HCLFLP and LCHFLP arms in the post-exercise (*p* = 0.041, denoted via the # Table 3) and overall (*p* = 0.036, denoted via the # Table 3) periods. Though there was a main effect identified in the overall CV between arms (HCLFLP: 7.2 ± 2.7, LCHFLP: 10.5 ± 4.9 LCLFHP: 11.8 ± 7.0%, *p* = 0.044) pairwise comparisons failed to identify the source of significance, but the greatest differences were between the HC and two alternative arms (HCLFLP vs. LCHFLP *p* = 0.099. HCLFLP vs. LCLFHP, *p* = 0.098).

#### 3.2.2. Plasma Lactate Responses

A main effect was found for time (F [1.432, 12.890] = 16.473, *p* < 0.001) but not diet (F [1.257, 11.312] = 2.042, *p* = 0.180). There were no differences between arms in PLa at any time point throughout the trial period (Figure 1, panel C). The Δ_Ex_ in PLa was also comparable between arms (HCLFLP: Δ + 0.74 ± 0.68, LCLFHP: Δ − 0.85 ± 0.79, LCHFLP: Δ + 0.50 ± 0.70 mmol/L, *p* = 0.419).

#### 3.2.3. Non-Esterified Fatty Acid Responses

A main effect was found for time (F [1.670, 15.027] = 16.475, *p* < 0.001) but not diet (F [2, 18] = 2.422, *p* = 0.117). However, pairwise comparisons of simple effects identified significance between the HCLFLP and LCHFLP diet arms (*p* = 0.027). Point concentrations of NEFA differed between the two arms at minutes 30 (*p* = 0.022) and 150 (*p* = 0.019), denoted via the # in Figure 1 panel D. In both the HCLFLP and LCHFLP arms, NEFA levels were elevated from their respective baseline concentrations at minute 150 (denoted via the black markers in Figure 1, panel D).

The Δ_Ex_ in NEFA was similar between arms (HCLFLP: Δ + 0.36 ± 0.28, LCLFHP: Δ + 0.38 ± 0.28, LCHFLP: Δ + 0.45 ± 0.32 mmol/L, *p* = 0.396). Relative to those measured at exercise onset (minute 90), NEFA concentrations were raised (Δ + 0.45 mmol/L) 30 min into cycling in the LCHFLP arm (patterned marker Figure 1 panel D). The rise fell outside of significance in the alternate two arms.

#### 3.2.4. Insulin Responses

A main effect was found for time (F [1.253, 12.526] = 6.013, *p* = 0.024) but not diet (F [2, 20] = 1.288, *p* = 0.298), with comparable insulin levels noted across the trial day (all *p* ≥ 0.05, Figure 1, panel E). Insulin levels had dropped from baseline values by minute 30 in the HCHFLP arm (*p* = 0038, denoted via the black marker in Figure 1, panel E). No other within-trial arm changes were observed. The Δ_Ex_ in insulin was similar between arms (HCLFLP: Δ + 0.65 ± 3.07, LCLFHP: Δ − 0.27 ± 3.50, LCHFLP: Δ + 0.04 ± 1.27 mU/L, *p* = 0.396).

#### 3.2.5. Glucagon Responses

Figure 1 panel F displays the glucagon concentrations across the trial period for all three dietary arms. There were no differences in glucagon concentrations between diets at any time point throughout the trial day (F [2, 20] = 1.3718, *p* = 0.493) with no obvious time effect (F [1, 2] = 2.412, *p* = 0.121), nor were there any signs of significant change in glucagon values from each arms respective baseline or pre-exercise values (all *p* ≥ 0.05). Δ_Ex_ in glucagon was similar between arms (HCLFLP: Δ + 2.75 ± 5.12, LCLFHP: Δ + 1.50 ± 4.38, LCHFLP: Δ + 3.25 ± 6.67 pmol/L, *p* = 0.690).

### 3.3. Cardiopulmonary and Calorimetric Data

Table 4 displays the cardiorespiratory parameters collected from the fasted rested period.

Table 5 displays the cardiorespiratory parameters collected from the 45 min moderate intensity continuous exercise session (~60% $\dot{V}$O_2peak_).

Table 6 displays the cardiorespiratory parameters collected from the rested post-exercise period_._

## 4. Discussion

This study compared the metabolic responses to fasted submaximal cycling in adults with T1D following seven days of adherence to diets differing in macronutrient composition. The major findings of this study are as follows: (1) Compared to a high-carbohydrate, low-fat diet, consuming a low-carbohydrate, high-fat one led to a ≈1.2-fold increase in the rate of fat oxidation (with a concurrent 0.8-fold decrease in the rate of carbohydrate oxidation) during exercise as well as an increased appearance of circulating free fatty acids shortly after cycling. (2) Glucose concentrations remained stable throughout exercise with no incidences of hypoglycaemia regardless of which diet had been adhered to in the seven days prior.

### 4.1. Substrate Availability and Fuel Oxidation

In the present study, the exercise bout was standardised at a constant workload (≈60% $\dot{V}$O_2peak_) for a fixed duration of 45 min after an overnight fast on each occasion. Implementation of this model was important not only in being able to discern potential adaptations in oxidation rates without the confounding effects of pre-exercise nutrition [8], but also because exercise intensity presides over dietary manipulation and substrate availability in determining the choice of fuel by the working muscle [10,30]. During each cycling session in this study, the stimulation of energy expenditure was identical on all three occasions equating to ≈336 kcals. In all arms, the relative contribution of energy derived from the oxidation of lipids outweighed that of carbohydrates (accounting for >50% of energy output within each arm). However, inter-arm comparisons showed that consuming a LCHFLP diet led to a ≈1.2-fold increase in the rate of fat oxidation, and concurrent 0.8-fold decrease in the rate of carbohydrate oxidation, during exercise compared to a HCLFLP diet.

The substrate shift findings of the present study align with work by others who have also demonstrated that the adoption of a low-carbohydrate, high-fat diet with a similar macronutrient compositional split to ours (i.e., >65% energy intake from fat, <20% energy intake from carbohydrate) by healthy well-trained cyclists over a short-term period (five days to two weeks) resulted in high rates of fat oxidation (1.7- to 2-fold) coupled with lower rates of carbohydrate oxidation during moderate intensity cycling exercise (60–70% $\dot{V}$O_2peak_) whilst fasting [13,31,32]. Whether the same phenomenon is observable in other exercise modalities (e.g., high intensity interval or resistance exercise) that evoke divergent effects on energy metabolism is deserving of investigation.

We have previously shown a much smaller contribution of lipid-to-energy output (≈36% of energy provision) when exercise of the same protocol used in this study, i.e., 45 min of steady state cycle ergometry at 60% $\dot{V}$O_2peak_, is undertaken by individuals with T1D in the acute, post-prandial period (90 min) following the ingestion of a low-glycaemic index carbohydrate load (≈60 g) with concomitant bolus insulin dose (≈5 units) [33]. Hence, exercise undertaken in the fasted state, particularly when combined with a low carbohydrate diet, may constitute an attractive model for facilitating fuel shifts in oxidative metabolism and increasing the capacity of the muscle to utilise fat whilst sparing glycogen stores. The integration of advanced techniques to assess intramuscular metabolic activity would be useful in corroborating these hypotheses whilst furthering our mechanistic understanding of dietary-induced metabolic adaptations to exercise.

In the overnight-fasted state the primary sources of oxidised fat during moderate intensity exercise are plasma FFA derived from adipose tissue lipolysis and intramuscular triglyceride [9,34]. Resting muscle blood flow is low (1–4 mL/min per 100 g) but increases linearly with exercise workload to levels some 10- to 15-fold higher than rest. Accordingly, the active muscle NEFA uptake, and subsequent oxidation, is tightly related to the rate of delivery (concentration*plasma flow) to the active muscle [9]. Fittingly, we saw elevated concentrations of NEFA at rest (before exercise) in the LCHFLP dietary arm, which progressively increased throughout the exercise session to levels that were substantially higher than those of the HCLFLP arm acutely after cessation. Though the exercise-induced increase in NEFA was comparable between arms (≈+0.4 mmol/L, [ a 2-fold increase]), the magnitude of intra-arm change towards the latter stages (from 30 min onwards) of cycling was only statistically significant in the LCHFLP arm. The progressive increases in NEFA concentrations over exercise resulted in peak concentrations in the acute post-exercise period (+15 min after exercise cessation) in all three arms (HCLFLP: 1.19 mmol/L, LCHFLP: 1.49 mmol/L, LCLFHP: 1.34 mmol/L) to levels in keeping with normative values (1–2 mmol/L) typically observed after exercise cessation as a result of continued stimulation of lipolysis despite an abrupt fall in muscle uptake [35]. Nevertheless, at this time point, NEFAs concentrations were proportionately higher in the LCHFLP arm compared to those measured in the HCLFLP one (by ≈22%).

The observed alterations in whole-body substrate oxidation with low-carbohydrate, high-fat diets may be attributed, in part, to alterations in substrate availability, as intramuscular triglyceride stores increase, and muscle glycogen concentrations decrease [11,34]. Though without muscle biopsy data in the present study we cannot corroborate any intramuscular adaptions, we did find that the HF diet elicited an increased dependence on lipid as a fuel source. Contextually, this may come with some potential advantages in those with T1D. The greater the sparing effect of limited endogenous carbohydrate reserves, the greater the scope for the prolongation of exercise. Longer exercise duration comes with a higher energetic output and, hence, a greater caloric expenditure with possible benefit from a weight management perspective. This is compounded by the fact that people with T1D often have to prematurely terminate and/or pause exercise due to the need to ingest exogenous carbohydrates (which in turn come with a caloric ‘cost’) to correct falling blood glucose levels. Of equal importance might be the preservation of endogenous carbohydrate stores for glucose homeostasis after exercise cessation, at a time when tissue sensitivity to insulin is raised and there is, therefore, a continued need to consider exogenous insulin therapy management to minimise the likelihood of late-onset, post-exercise hypoglycaemia [5,36,37].

### 4.2. Glucose Responses

In all three arms, glucose levels throughout exercise remained stable and comparable to those taken immediately prior to commencing cycling, changing by less than 0.75 mmol/L (direction aside) in every trial. Furthermore, there were no incidences of hypoglycaemia (<3.9 mmol/L) in any trial arm with glucose levels never breaching <4.0 mmol/L in any participant. This is in notable contrast to studies we have previously conducted in which the exact same exercise model (i.e., moderate intensity continuous cycling at 60% $\dot{V}$O_2peak_) was undertaken but within the acute post-prandial window, i.e., 90 min after ingesting 0.75g carbohydrates per kg body mass with bolus insulin. Under these conditions pooled analyses on our data collected from two separate studies implementing this methodology show consistent and precipitous drops in glucose (by ≈−3 mmol/L, ranging from −3.0 to 3.9 mmol/L) over cycling with a high prevalence of hypoglycaemia during and/or acutely after exercise [4,38]. Considering hypoglycaemia and loss of glycaemic regulation constitute leading barriers to regular exercise engagement in those with T1D [39], conducting exercise in the morning whilst fasted may be worthy of consideration for cohorts who struggle with exercise management, irrespective of diet.

Interestingly, the LCLFHP arm was the only one in which the grouped mean glucose dropped, rather than increased, over exercise and to an order of magnitude that made it differ from that of the HCLFLP arm (Δ_Ex_ −0.44 vs. +0.72 mmol/L, respectively, *p* = 0.044). Indeed, mean PG concentrations were raised throughout the exercise, post-exercise, and overall time periods in the HCLFLP arm relative to the LCLFHP arm. Interestingly, during the post-exercise period, the elevated glycaemic profile of the HCLFLP arm resulted in it, also differing from the LCHFLP arm in both TIR and TAR (both by ≈36%). These distinct differences led to the high-carbohydrate arm deviating from the two low-carbohydrate arms in TIR and TAR by at least 30% (↓ and ↑, respectively) over the entire trial period. Worth highlighting is the lack of discernible difference in any glycaemic parameter between the two LC arms, which also had comparable responses in all other metabolic and hormonal biomarkers throughout.

The findings of the current study provide supported extension of those produced by our laboratory group during which we showed favourable sensor glucose levels during the one-week dietary intervention after the adoption of the two low-carbohydrate diets in question relative to the high-carbohydrate arm [26]. Importantly, the observed effect of carbohydrate restriction on glycaemia was consistent regardless of whether supplementary energy was substituted by that of fat or protein [26].

An important caveat to any dietary intervention is that is must be individualised and most importantly *enjoyed* by the person to whom it is prescribed. We would therefore like to emphasise that the data herein are less about promoting one diet per se but rather detailing the effects of alterations in macronutrient composition on energy metabolism during exercise that is performed under fasted conditions by people with T1D.

Strengths, limitations, and perspectives for future research directions.

This study is not without its limitations. Admittedly, the small sample size was rather homogenous and the duration of the diet intervention period relatively short. Hence, larger and longer-lasting trials that provide a more heterogenous population with protracted exposure to each diet are needed to corroborate our findings. Furthermore, explorations as to whether these results can be extrapolated to different exercise modalities (e.g., high-intensity interval or resistance exercise training) or evenly between sexes would be helpful in informing a more rounded approach to nutritional counselling in exercise management. Nevertheless, the high degree of methodological standardisation and rigour incorporated in this study provides assurance of the quality of data which we hope can be used as a foundational basis for incentivising future research efforts.

## 5. Conclusions

In summary, we found that consuming a low-carbohydrate, high-fat, low-protein diet over seven days shifted patterns of fuel utilisation towards an increased utilisation of lipid and decreased reliance of carbohydrate metabolism during moderate intensity continuous exercise. Despite these shifts, plasma glucose concentrations remained stable and unchanged from the respective pre-exercise values throughout cycling in all three dietary arms. Such information serves to remind of the potentiality of nutrition in modulating exercise metabolism in individuals with T1D.

## Figures and Tables

**Table 1:** Diet compositions (in grams and energy percentages) for a 70 kg person with a calorie need of 30 Kcal/kg/day. HCLFLP; high carbohydrate, low fat, low protein, LCHFLP; low carbohydrate, high fat, low protein, and LCLFHP; low carbohydrate, low fat, high protein.

Main Macronutrient Composition of the Diet	Carbohydrates	Fat	Protein
**High carbohydrate (HCLFLP)**	250 g (48%)	78 g (33%)	98 g (19%)
**High fat (LCHFLP)**	100 g (19%)	145 g (62%)	98 g (19%)
**High protein (LCLFHP)**	100 g (19%)	132 g (57%)	126 g (24%)

**Table 2:** Baseline characteristics of study participants. BMI: body mass index. HbA1c: Haemoglobin A1C. CHO: carbohydrates. SG: sensor glucose. CV: coefficient of variation. TBR: time spent with sensor glucose values below the target range (<3.9 mmol·L^−1^). TIR: time spent with sensor glucose values within the target range (3–9 to 10.0 mmol·L^−1^). TAR: time spent with sensor glucose values above the target range (>10.0 mmol·L^−1^).

Characteristic	Mean ± SD	Range (Min–Max)
Age (years)	46 ± 15	48 (22–70)
BMI (kg·m^−2^)	27.0 ± 4.3	14.6 (21.3–35.9)
HbA1c (%)	7.3 ± 0.7	2.8 (5.5–8.3)
HbA1c (mmol·mol^−1^)	55.9 ± 7.8	30.0 (37.0–67.9)
Diabetes duration (years)	29 ± 15	41 (11–52)
Age of diabetes onset (years)	17 ± 11	39 (4–43)
Total daily insulin dose (U)	45.4 ± 20.0	108 (18.4–127.0)
Total daily insulin dose (U·kg^−1^)	0.5 ± 0.2	0.7 (0.3–1.0)
Total daily basal insulin dose (percent of total)	48.4 ± 9.0	29.0 (34.9–63.9)
Total daily bolus insulin dose (percent of total)	51.6 ± 9.0	29.3 (36.1–65.4)
Average daily CHO intake (g)	160 ± 66	201 (62–263)
Average 14-day mean SG (mmol·L^−1^)	8.9 ± 0.9	3.0 (7.0–10.0)
Average 14-day SG CV (%)	37.9 ± 4.5	17.0 (29.0–46.0)
Average 14-day SG TBR (%)	10.8 ± 16.6	46 (0.0–46.0)
Average 14-day SG TIR (%)	61.1 ± 12.3	44.0 (39.0–83.0)
Average 14-day SG TAR (%)	28.2 ± 18.3	60.0 (1.0–61.0)
Systolic blood pressure (mmHg)	142 ± 9	24 (131–155)
Diastolic blood pressure (mmHg)	86 ± 6	23 (71–94)
Resting heart rate (bpm)	68 ± 12	43 (52–95)
$\dot{V}$O_2peak_ (L·min^−1^)	3.0 ± 1.1	3.1 (1.5–4.6)
$\dot{V}$O_2peak_ (mL·min·kg^−1^)	34.8 ± 10.9	36.0 (21.1–57.1)
Power_peak_ (L·min^−1^)	256 ± 84	225 (155–380)
Power_peak_ (Watts·kg^−1^)	3.1 ± 0.9	2.8 (1.9–4.7)

**Table 3:** Glycaemic parameters during each pre-defined time-period on experimental trial days. PG: plasma glucose. TBR: time spent with plasma glucose below the target range (<3.9 mmol/L). TIR: time spent with plasma glucose levels within the target range (3.9–10 mmol/L). TAR: time spent with plasma glucose above the target range (>10 mmol/L). CV: coefficient of variation. * *p* ≤ 0.05 between HCLFLP and LCLFHP. ^#^ *p* ≤ 0.05 between HCLFLP and LCHFLP.

Glycaemic Parameter	HCLFLP	LCHFLP	LCLFHP	*p*-Value
**Pre-exercise period_(0min to 60min)_**				
Mean PG (mmol/L)	9.5 ± 2.1	8.2 ± 0.9	8.1 ± 1.6	0.052
SD PG (mmol/L)	0.4 ± 0.2	0.6 ± 0.4	0.6 ± 0.3	0.226
CV PG (%)	4.6 ± 2.0	7.4 ± 5.0	7.9 ± 4.7	0.087
TBR PG (%)	-	-	-	-
TIR PG (%)	66.7 ± 46.2	95.0 ± 17.3	86.7 ± 26.1	0.078
TAR PG (%)	33.3 ± 46.2	5.0 ± 17.3	13.3 ± 26.1	0.078
**Exercise period_(90min to 135min)_**				
Mean PG (mmol/L)	10.1 ± 2.8	8.5 ± 1.9	7.6 ± 1.3	0.004 *
SD PG (mmol/L)	0.5 ± 0.2	0.5 ± 0.3	0.5 ± 0.3	0.947
CV PG (%)	4.7 ± 1.6	5.5 ± 2.2	6.3 ± 4.8	0.501
TBR PG (%)	-	-	-	*-*
TIR PG (%)	59.2 ± 47.2	88.3 ± 30.1	99.2 ± 2.9	0.006 *
TAR PG (%)	40.8 ± 47.2	11.7 ± 30.1	0.8 ± 2.9	0.006 *
**Post-exercise period_(140min to 225min)_**				
Mean PG (mmol/L)	10.7 ± 3.2	8.8 ± 2.8	7.5 ± 1.8	0.003 *
SD PG (mmol/L)	0.4 ± 0.2	0.3 ± 0.1	0.2 ± 0.1	0.040
CV PG (%)	3.5 ± 1.8	3.4 ± 1.4	3.4 ± 1.1	0.973
TBR PG (%)	-	-	-	-
TIR PG (%)	44.8 ± 44.7	81.3 ± 38.3	96.9 ± 10.8	0.002 *^#^
TAR PG (%)	55.2 ± 44.7	18.8 ± 38.3	3.1 ± 10.8	0.002 *^#^
**Overall period_(0min to 225min)_**				
Mean PG (mmol/L)	10.2 ± 2.7	8.6 ± 1.9	7.7 ± 1.3	0.004 *
SD PG (mmol/L)	0.7 ± 0.4	0.9 ± 0.5	0.9 ± 0.4	0.462
CV PG (%)	7.2 ± 2.7	10.5 ± 4.9	11.8 ± 7.0	0.044 *^#^
TBR PG (%)	-	-	-	*-*
TIR PG (%)	55.6 ± 43.9	87.3 ± 28.7	95.2 ± 7.9	0.003 *^#^
TAR PG (%)	44.4 ± 43.9	12.7 ± 28.7	4.8 ± 7.9	0.003 *^#^

**Table 4:** Cardiorespiratory parameters collected from the fasted morning rested period. $\dot{V}$O_2_: Volume of Oxygen uptake. $\dot{V}$CO_2_: Volume of carbon dioxide production. RER: Respiratory exchange rate (ratio of $\dot{V}$CO_2_ divided by $\dot{V}$O_2_). O_2_ pulse: Oxygen pulse ($\dot{V}$O_2_/HR). Kcals: Kilocalories. KJ: Kilojoules.

Parameter	HCLFLP	LCLFHP	LCHFLP	*p* Value
Heart rate (bpm)	60 ± 10	59 ± 7	61 ± 8	0.289
$\dot{V}$O_2_ (L/min)	0.3 ± 0.1	0.3 ± 0.1	0.3 ± 0.1	0.858
$\dot{V}$CO_2_ (L/min)	0.2 ± 0.0	0.2 ± 0.1	0.2 ± 0.1	0.747
RER	0.8 ± 0.1	0.8 ± 0.0	0.8 ± 0.0	0.203
O_2_ pulse (mL/min)	5.1 ± 1.5	5.2 ± 1.4	5.0 ± 1.1	0.465
Carbohydrate oxidation (g/min)	0.13 ± 0.08	0.12 ± 0.04	0.10 ± 0.04	0.292
Carbohydrate oxidation (% total energy)	36.3 ± 19.9	32.1 ± 6.9	27.9 ± 7.1	0.205
Lipid oxidation (g/min)	0.11 ± 0.05	0.12 ± 0.03	0.12 ± 0.02	0.364
Lipid oxidation (% total energy)	64.7 ± 19.9	67.9 ± 6.9	72.1 ± 7.1	0.205
Total energy (Kcals)	22.6 ± 4.9	23.2 ± 5.7	22.1 ± 4.4	0.442
Total energy (KJ)	94.4 ± 20.6	97.0 ± 23.6	92.6 ± 18.6	0.441

**Table 5:** Cardiorespiratory parameters collected from the 45 min moderate intensity continuous exercise session. $\dot{V}$O_2_: Volume of Oxygen uptake. $\dot{V}$CO_2_: Volume of carbon dioxide production. RER: Respiratory exchange rate (ratio of $\dot{V}$CO_2_ divided by $\dot{V}$O_2_). O_2_ pulse: Oxygen pulse ($\dot{V}$O_2_/HR). Kcals: Kilocalories. KJ: Kilojoules. ^#^ *p* ≤ 0.05 between HCLFLP and LCHFLP.

Parameter	HCLFLP	LCLFHP	LCHFLP	*p* Value
Exercise duration (mins)	43.9 ± 3.4	44.9 ± 0.3	44.8 ± 0.9	0.427
Load (watts)	95.7 ± 38.9	96.3 ± 37.9	95.8 ± 39.0	0.300
Heart rate (bpm)	119 ± 10	119 ± 13	122 ± 12	0.343
$\dot{V}$O_2_ (L/min)	1.6 ± 0.5	1.6 ± 0.5	1.6 ± 0.5	0.260
$\dot{V}$CO_2_ (L/min)	1.3 ± 0.4	1.3 ± 0.4	1.3 ± 0.4	0.257
RER	0.83 ± 0.03	0.81 ± 0.04	0.80 ± 0.04	0.048 ^#^
O_2_ pulse (mL/min)	12.9 ± 4.1	12.7 ± 3.5	12.8 ± 4.4	0.810
Carbohydrate oxidation (g/min)	0.87 ± 0.27	0.75 ± 0.37	0.70 ± 0.36	0.089
Carbohydrate oxidation (% total energy)	46.5 ± 9.2	39.6 ± 14.4	35.7 ± 14.6	0.039 ^#^
Lipid oxidation (g/min)	0.46 ± 0.18	0.50 ± 0.20	0.54 ± 0.21	0.113
Lipid oxidation (% total energy)	53.5 ± 9.2	60.4 ± 14.4	64.3 ± 14.6	0.039 ^#^
Total energy (Kcals)	335.8 ± 111.4	337.9 ± 107.1	343.0 ± 112.6	0.552
Total energy (KJ)	1403.5 ± 465.8	1412.2 ± 447.6	1433.9 ± 470.7	0.552

**Table 6:** Cardiorespiratory parameters collected from the rested post-exercise period. $\dot{V}$O_2_: Volume of Oxygen uptake. $\dot{V}$CO_2_: Volume of carbon dioxide production. RER: Respiratory exchange rate (ratio of $\dot{V}$CO_2_ divided by $\dot{V}$O_2_). O_2_ pulse: Oxygen pulse ($\dot{V}$O_2_/HR). Kcals: Kilocalories. KJ: Kilojoules.

Parameter	HCLFLP	LCLFHP	LCHFLP	*p* Value
Heart rate (bpm)	65 ± 10	61 ± 7	64 ± 10	0.231
$\dot{V}$O_2_ (L/min)	0.3 ± 0.1	0.3 ± 0.1	0.3 ± 0.1	0.842
$\dot{V}$CO_2_ (L/min)	0.2 ± 0.0	0.2 ± 0.1	0.2 ± 0.1	0.702
RER	0.75 ± 0.05	0.72 ± 0.04	0.74 ± 0.05	0.115
O_2_ pulse (mL/min)	5.0 ± 1.4	5.2 ± 1.5	5.0 ± 1.5	0.692
Carbohydrate oxidation (g/min)	0.12 ± 0.05	0.10 ± 0.04	0.11 ± 0.05	0.214
Carbohydrate oxidation (% total energy)	30.0 ± 14.2	22.8 ± 8.3	26.8 ± 9.9	0.099
Lipid oxidation (g/min)	0.13 ± 0.05	0.15 ± 0.06	0.14 ± 0.05	0.377
Lipid oxidation (% total energy)	70.0 ± 14.2	77.2 ± 8.3	73.2 ± 9.9	0.099
Total energy (Kcals)	25.2 ± 6.8	25.8 ± 9.1	25.1 ± 7.5	0.890
Total energy (KJ)	105.3 ± 28.3	108.0 ± 38.2	104.9 ± 31.2	0.889

## Data Availability

The original contributions presented in this study are included in the article. Further inquiries can be directed to the corresponding authors.
